# Supplementary material for: Pyruvate Dehydrogenase Kinase 1 inhibition mediated oxidative phosphorylation enhancement in cartilage promotes osteoarthritis progression
Source: BMC Musculoskelet Disord. 2023 Jul 20;24:597. doi: 10.1186/s12891-023-06585-6 (PMC10357736; doi:10.1186/s12891-023-06585-6)
Supplement: Supplementary file 1 — Additional file 1. [file 12891_2023_6585_MOESM1_ESM.zip › MF.Gsea.1653627316242/GOMF_MRNA_5_UTR_BINDING.html]

Details for gene set GOMF\_MRNA\_5\_UTR\_BINDING[GSEA]

|  || Dataset | 55457\_collapsed\_to\_symbols.cls.cls #OA\_versus\_normal.cls.cls #OA\_versus\_normal\_repos |
| Phenotype | cls.cls#OA\_versus\_normal\_repos |
| Upregulated in class | normal |
| GeneSet | GOMF\_MRNA\_5\_UTR\_BINDING |
| Enrichment Score (ES) | -0.5637514 |
| Normalized Enrichment Score (NES) | -1.7949525 |
| Nominal p-value | 0.003478261 |
| FDR q-value | 0.08095502 |
| FWER p-Value | 0.567 |
Table: GSEA Results Summary

  

Fig 1: Enrichment plot: GOMF\_MRNA\_5\_UTR\_BINDING      
 Profile of the Running ES Score & Positions of GeneSet Members on the Rank Ordered List

  

| SYMBOL | TITLE | RANK IN GENE LIST | RANK METRIC SCORE | RUNNING ES | CORE ENRICHMENT || 1 | MRPS11 | mitochondrial ribosomal protein S11 [Source:HGNC Symbol;Acc:HGNC:14050] | 1305 | 0.457 | -0.0537 | No |
| 2 | SHMT2 | serine hydroxymethyltransferase 2 [Source:HGNC Symbol;Acc:HGNC:10852] | 1518 | 0.426 | -0.0258 | No |
| 3 | IGF2BP3 | insulin like growth factor 2 mRNA binding protein 3 [Source:HGNC Symbol;Acc:HGNC:28868] | 2770 | 0.273 | -0.0945 | No |
| 4 | MYH10 | myosin heavy chain 10 [Source:HGNC Symbol;Acc:HGNC:7568] | 4169 | 0.141 | -0.1884 | No |
| 5 | LARP6 | "La ribonucleoprotein 6, translational regulator [Source:HGNC Symbol;Acc:HGNC:24012]" | 4798 | 0.087 | -0.2280 | No |
| 6 | CCT5 | chaperonin containing TCP1 subunit 5 [Source:HGNC Symbol;Acc:HGNC:1618] | 7690 | -0.134 | -0.4386 | No |
| 7 | FMR1 | FMRP translational regulator 1 [Source:HGNC Symbol;Acc:HGNC:3775] | 7944 | -0.151 | -0.4425 | No |
| 8 | RSL1D1 | ribosomal L1 domain containing 1 [Source:HGNC Symbol;Acc:HGNC:24534] | 8286 | -0.177 | -0.4505 | No |
| 9 | SHMT1 | serine hydroxymethyltransferase 1 [Source:HGNC Symbol;Acc:HGNC:10850] | 9727 | -0.294 | -0.5317 | Yes |
| 10 | RARA | retinoic acid receptor alpha [Source:HGNC Symbol;Acc:HGNC:9864] | 10141 | -0.335 | -0.5289 | Yes |
| 11 | RPL5 | ribosomal protein L5 [Source:HGNC Symbol;Acc:HGNC:10360] | 10201 | -0.341 | -0.4979 | Yes |
| 12 | RPS14 | ribosomal protein S14 [Source:HGNC Symbol;Acc:HGNC:10387] | 10558 | -0.379 | -0.4861 | Yes |
| 13 | RPS7 | ribosomal protein S7 [Source:HGNC Symbol;Acc:HGNC:10440] | 10608 | -0.384 | -0.4499 | Yes |
| 14 | GNL3 | G protein nucleolar 3 [Source:HGNC Symbol;Acc:HGNC:29931] | 10907 | -0.418 | -0.4295 | Yes |
| 15 | LARP1 | "La ribonucleoprotein 1, translational regulator [Source:HGNC Symbol;Acc:HGNC:29531]" | 10964 | -0.425 | -0.3895 | Yes |
| 16 | SYNCRIP | synaptotagmin binding cytoplasmic RNA interacting protein [Source:HGNC Symbol;Acc:HGNC:16918] | 11182 | -0.454 | -0.3590 | Yes |
| 17 | IGF2BP2 | insulin like growth factor 2 mRNA binding protein 2 [Source:HGNC Symbol;Acc:HGNC:28867] | 12244 | -0.657 | -0.3730 | Yes |
| 18 | NCL | nucleolin [Source:HGNC Symbol;Acc:HGNC:7667] | 12255 | -0.662 | -0.3048 | Yes |
| 19 | RPS13 | ribosomal protein S13 [Source:HGNC Symbol;Acc:HGNC:10386] | 12334 | -0.687 | -0.2393 | Yes |
| 20 | RPL41 | ribosomal protein L41 [Source:HGNC Symbol;Acc:HGNC:10354] | 12674 | -0.847 | -0.1774 | Yes |
| 21 | RPS3A | ribosomal protein S3A [Source:HGNC Symbol;Acc:HGNC:10421] | 12758 | -0.921 | -0.0879 | Yes |
| 22 | DDX3X | DEAD-box helicase 3 X-linked [Source:HGNC Symbol;Acc:HGNC:2745] | 12788 | -0.949 | 0.0087 | Yes |
Table: GSEA details [plain text format]

  

Fig 2: GOMF\_MRNA\_5\_UTR\_BINDING      
 Blue-Pink O' Gram in the Space of the Analyzed GeneSet

  

Fig 3: GOMF\_MRNA\_5\_UTR\_BINDING: Random ES distribution      
 Gene set null distribution of ES for **GOMF\_MRNA\_5\_UTR\_BINDING**
